# Underestimation of foraging behaviour by standard field methods in malaria vector mosquitoes in southern Africa

**DOI:** 10.1186/s12936-014-0527-9

**Published:** 2015-01-21

**Authors:** Smita Das, Tyler C Henning, Limonty Simubali, Harry Hamapumbu, Lukwa Nzira, Edmore Mamini, Aramu Makuwaza, Mbanga Muleba, Douglas E Norris, Jennifer C Stevenson, for the Southern Africa ICEMR Team

**Affiliations:** The W. Harry Feinstone Department of Molecular Microbiology and Immunology, The Johns Hopkins Malaria Research Institute, Johns Hopkins University Bloomberg School of Public Health, Baltimore, MD USA; Macha Research Trust, P.O. Box 630166, Choma, Zambia; National Institute of Health Research, P.O. Box 573, Harare, Zimbabwe; Biomedical Research Training Institute, Harare, Zimbabwe; Tropical Disease Research Centre, Ndola, Zambia

**Keywords:** Malaria, *Anopheles* mosquitoes, Human blood index, Zambia, Zimbabwe, ICEMR

## Abstract

**Background:**

Defining the anopheline mosquito vectors and their foraging behaviour in malaria endemic areas is crucial for disease control and surveillance. The standard protocol for molecular identification of host blood meals in mosquitoes is to morphologically identify fed mosquitoes and then perform polymerase chain reaction (PCR), precipitin tests, or ELISA assays. The purpose of this study was to determine the extent to which the feeding rate and human blood indices (HBIs) of malaria vectors were underestimated when molecular confirmation by PCR was performed on both visually fed and unfed mosquitoes.

**Methods:**

In association with the Southern Africa International Centers of Excellence in Malaria Research (ICEMR), mosquito collections were performed at three sites: Choma district in southern Zambia, Nchelenge district in northern Zambia, and Mutasa district in eastern Zimbabwe. All anophelines were classified visually as fed or unfed, and tested for blood meal species using PCR methods. The HBIs of visually fed mosquitoes were compared to the HBIs of overall PCR confirmed fed mosquitoes by Pearson’s Chi-Square Test of Independence.

**Results:**

The mosquito collections consisted of *Anopheles arabiensis* from Choma, *Anopheles funestus s.s.*, *Anopheles gambiae s.s*. and *Anopheles leesoni* from Nchelenge, and *An. funestus s.s.* and *An. leesoni* from Mutasa. The malaria vectors at all three sites had large human blood indices (HBI) suggesting high anthropophily. When only visually fed mosquitoes tested by PCR for blood meal species were compared to testing those classified as both visually fed and unfed mosquitoes, it was found that the proportion blooded was underestimated by up to 18.7%. For most *Anopheles* species at each site, there was a statistically significant relationship (*P* < 0.05) between the HBIs of visually fed mosquitoes and that of the overall PCR confirmed fed mosquitoes.

**Conclusion:**

The impact on HBI of analysing both visually fed and unfed mosquitoes varied from site to site. This discrepancy may be due to partial blood feeding behaviour by mosquitoes, digestion of blood meals, sample condition, and/or expertise of entomology field staff. It is important to perform molecular testing on all mosquitoes to accurately characterize vector feeding behaviour and develop interventions in malaria endemic areas.

## Background

Malaria is a significant public health problem in Africa, killing hundreds of thousands of children annually [1]. In sub-Saharan Africa, *Plasmodium falciparum* malaria is the most common malaria parasite and is transmitted by mosquito species belonging to the *Anopheles* genus. The extent of vector-host association is one of the most important factors in predicting vectorial capacity [2,3] and forms the basis for the Ross-MacDonald model and other contemporary models that estimate malaria transmission intensity [4-7]. The human blood index (HBI), or the proportion of blood meals taken on humans by mosquitoes, varies dramatically even within a single taxon, across localities and between seasons [5], and reflects differences in intrinsic host preferences, host availability, and accessibility [8-12]. The HBI of malaria vectors is used to determine anthropophily, changes in feeding behaviour, and even multiple blood feeding frequency [10,13-16]. Host preference studies have also been used to monitor the effectiveness of vector control programmes by observing a reduction in blood feeding behaviour, and have even served as evidence of control failure [17-20]. Additionally, the counts of human blood fed mosquitoes from pyrethrum spray catches (PSCs) have been used as a correlate of biting rate in the estimation of the entomological inoculation rate (EIR), or the number of infectious bites per person per time period. Measurement of EIRs gives an estimation of transmission intensity in an area [21] and can be used to determine the contribution of each vector species to malaria transmission in a particular locale [22,23]. Variations in EIRs over time and space are, therefore, often used to assess effectiveness of control and identify malaria foci [24].

In the field, one of the first steps in ascertaining the blood meal host is to visually identify and separate collected mosquitoes based on species and feeding status. The mosquitoes that appear morphologically blooded are labelled as “fed” and it is these samples that are usually separated for blood meal analysis for host species identification or simply counted if exclusive host association is assumed or capacity for molecular analysis is unavailable. However, the possibility remains that some collected mosquitoes may have taken a small or partial blood meal or may have partially digested the blood and are indeed fed, but morphologically appear “unfed”. Most importantly, these mosquitoes represent vectors that have bitten a host and, therefore, could have potentially transmitted pathogens, but have evaded the “fed” count during field investigations. By not evaluating these mosquitoes for blood meal host, the blood feeding frequency and EIR may be significantly underestimated and HBI miscalculated leading to inaccurate interpretations of vector foraging behaviour, parasite transmission, and malaria control.

In this study, mosquitoes were collected in three distinct epidemiological areas in southern with the aim to estimate the disparity in morphological and molecular assessments of anopheline feeding status.

## Methods

### Study area

**Figure 1:**
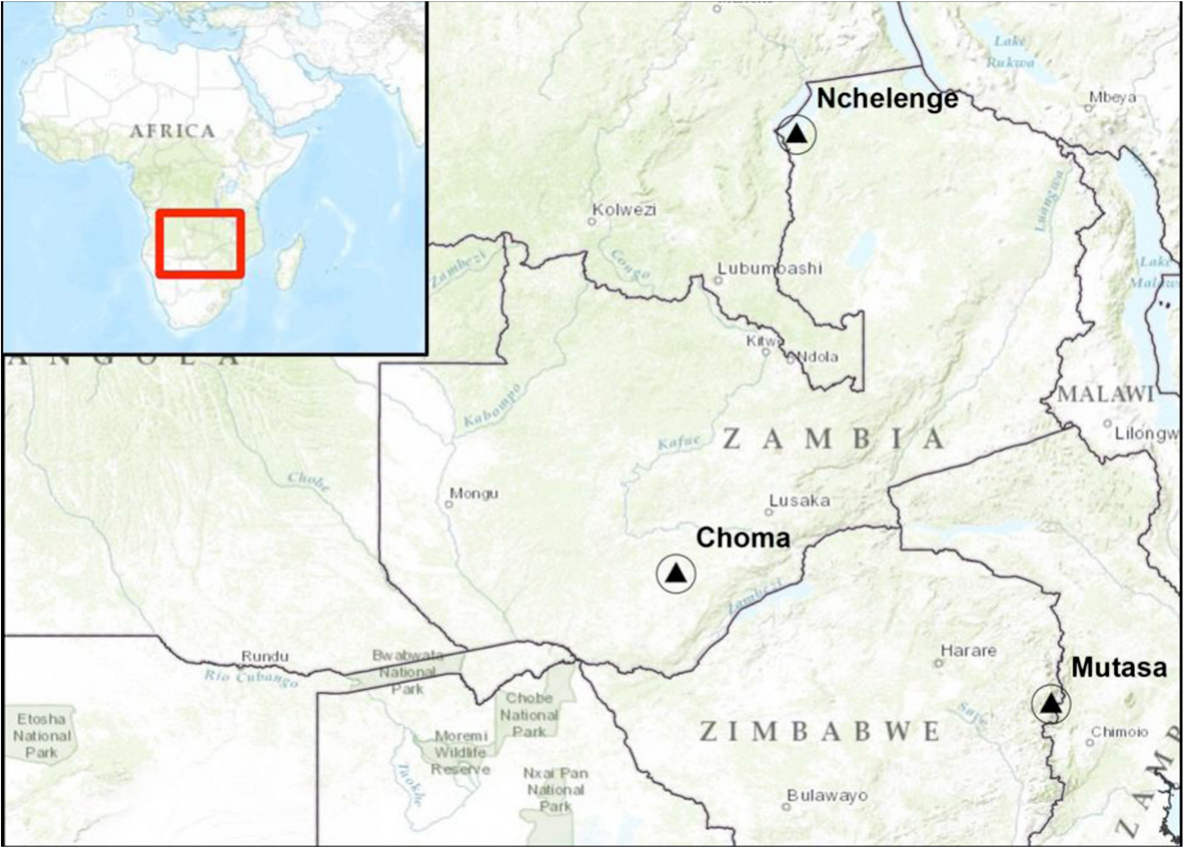
**Southern Africa ICEMR sites: Choma District, southern Zambia, Nchelenge District, northern Zambia, and Mutasa District, eastern Zimbabwe.**

These studies were carried out in association with the Johns Hopkins Southern Africa International Centers for Excellence in Malaria Research (ICEMR) project at three field sites: Choma district, southern Zambia (16.39292°S, 26.79061°E), Nchelenge district, northern Zambia (9° 19.115′S, 28° 45.070′E), and Mutasa district, eastern Zimbabwe (18° 23.161′S, 32° 59.946′E) (See Figure 1) [25].

### Choma district

In Choma district, collections were done within the catchment area of the Macha Mission Hospital, approximately 65 kilometres northeast from Choma town, Southern Province at a mean altitude of 1,100 metres above sea level. Extensive malaria entomological and epidemiological studies have been conducted in this area since 2003 [25]. This area consists of mainly scrub bush land interspersed with seasonal streams (Miombo woodland) and the population consists of mainly cattle herders and subsistence farmers. There is a single rainy season each year (November to May), followed by a cool dry season (May to August) and a hot dry season (August to November). Vector control in the area relies on the use of long lasting insecticide-treated nets (LLINs). Household ownership is estimated to be more than 90% and usage greater than 75% for all age groups (unpublished data). Malaria transmission at this site is restricted to the rainy season. Households were randomly selected from a grid overlaid on satellite imagery and were either assigned to a longitudinal cohort of houses followed every other month or for cross-sectional studies samples in the interim months.

### Nchelenge district

The field site in Nchelenge district, Luapula Province borders the Democratic Republic of Congo and lies along Lake Mweru. The area is located at a mean elevation of 807 metres above sea level in a marsh ecotype. The majority of the population in this area participates in subsistence farming and fishing. The seasons closely follow that of Choma District, although malaria transmission occurs year-round with a seasonal peak during the rains. Current vector control in this area includes LLIN distribution and indoor residual spraying (IRS) with bendiocarb and in the past pyrethroids. Net ownership and usage amongst study households is lower than that of Macha, with approximately 70% of households owning LLINs and usage across all age groups of approximately 50% (unpublished data). Longitudinal and cross-sectional households that were already enrolled in the ICEMR programme and were also located within two defined 1 km^2^ grids along both Lake Mweru and Kenani Stream were chosen for mosquito sampling.

### Mutasa district

The study site in Mutasa district, Manicaland Province, Zimbabwe bordering Mozambique is an area marked by broad elevation changes, with a range of approximately 600 to 1,300 metres above sea level. The majority of the population lives in Honde Valley, which has an average elevation of 900 metres above sea level. Subsistence farming occurs along streams and rivers, but there are several large tea estates within the district. Malaria transmission is seasonal, occurring most intensively during the wet season between November and April. Cool dry and hot dry seasons occur similarly to the study sites in Zambia. This area is targeted annually for IRS, and LLIN ownership is estimated at 88% and usage across all age groups at 70% for the study households (unpublished data). Mosquito collections took place in longitudinal and cross-sectional households that were randomly selected from 1-km^2^ grids similar to the other sites.

### Mosquito collection and handling

Field collections took place from January 2012-December 2013 in Macha, March-April 2012 in Nchelenge, and December 2012-February 2013 in Mutasa. Mosquitoes were collected from consenting households using Center for Disease Control miniature light traps (CDC LTs; John W. Hock Ltd, Gainesville, FL, USA) at all sites, and additionally by PSCs in Nchelenge and Mutasa. Collection methods were approved by the Johns Hopkins Bloomberg School of Public Health IRB (#00003467) and in Zambia (TDRC/ERC/2010/14/11) and Zimbabwe (BRTI AP102/11). CDC LTs were hung indoors next to sleeping persons, approximately 1.5 m above the floor, and would typically run from 6:00pm to 6:00am. PSCs were performed in the morning (6:00am-10:00am) in selected households, where white sheets were placed on the floors and an aerosol insecticide (100% synthetic pyrethroid) was applied towards the ceiling, eaves, and walls. After approximately 15 minutes, the sheets were taken out of each household and knocked down mosquitoes were collected.

### Visual classification of bloodfed status

All collected mosquitoes were killed by freezing. Using a dissecting microscope, female anopheline mosquitoes were morphologically identified to species (both vectors and non-vectors) using standard keys [13] and visually classified to feeding (abdominal) status (“fed” or “unfed”). Each mosquito was placed individually into a labelled 0.6 mL microcentrifuge tube containing silica gel desiccant and cotton wool, and stored either at room temperature or frozen at -20°C until laboratory processing, which took place at both the Johns Hopkins University Bloomberg School of Public Health in Baltimore, Maryland and the Macha Research Trust in Macha, Zambia.

### Classification of blood fed status by DNA techniques

The head and thoraces of all anopheline mosquitoes were separated from the abdomen of each mosquito and DNA extraction of the abdomens was performed using a modified salt extraction [26]. Morphological identification of anopheline mosquitoes was confirmed using a PCR specific for members of the *An. gambiae* complex or *An. funestus* complex [27,28]. All specimens collected in Nchelenge and Mutasa were tested for blood meal species by PCR whilst in Macha only those determined to be the vector *An. arabiensis* were analysed due to the large number of specimens collected over the 2-year period. Specimens were tested using the Kent et al. multiplex PCR, which differentiates potential mammal host bloods through amplification of the *cytochrome b* gene of the mitochondrial genome producing a range of species-specific bands from 132 to 680 base pairs [26]. Samples that did not amplify a band(s) for blood meal host were then tested with a more sensitive PCR and restriction fragment length polymorphism (RFLP) assay [29]. In brief, the PCR technique described by Fornadel *et al.* [29] was used to amplify a 98 base pair region from the *cytochrome b* gene of the mitochondrial genome of the mammalian host, followed by a restriction enzyme digest that is specific to that animal host.

### Statistical analysis

The visual status of the mosquito abdomen and overall PCR confirmed feeding status for each vector species in each field site were compared and analysed by Pearson Chi-Square Test of Independence using STATA version 11. A *P* value less than 0.05 was considered statistically significant.

## Results

### Composition of *Anopheles* species

#### Choma district

From January 2012 to December 2013, 643 female *An. arabiensis* were collected from 113 traps across 69 different households in Choma district. All collected anophelines had their morphological identities confirmed by molecular methods, of which *An. arabiensis* comprised 67%.

#### Nchelenge district

From March-April 2012 in Nchelenge district, 411 *Anopheles* were collected from 98 CDC light traps and 264 PSCs from 31 households and morphological identity was confirmed by PCR analysis. *Anopheles funestus s.s.* accounted for 83.4% of the total collection followed by *An gambiae s.s.* (8.8%) and *An. leesoni* (7.8%).

#### Mutasa district

From December 2012-February 2013, 84 *Anopheles* were collected in Mutasa district from 43 CDC light traps and 14 PSCs from 13 households. Morphological identifications in the field were confirmed by molecular methods. The collection was composed of 97.6% *An. funestus s.s.* and 2.4% *An. leesoni*.

#### Determination of blood feeding frequency, blood meal source, and HBI for visually fed anophelines Choma district

In the collection, 11.7% (75/643) of *An. arabiensis* were classified visually as fed and of those 75, 48 (64%) were confirmed by both the Kent and Fornadel PCR, giving a feeding rate of 7.5% (see Table 1). Of the 48 blood fed confirmed *An. arabiensis*, 46 had fed on humans or mixed human/animal blood meal to give an HBI of 0.96. One of these specimens was found to have a mixed blood meal of human and goat.

**Table 1:** **Abdominal status and human blood indices (HBI) determined by molecular assays of visually fed and unfed anophelines at three field sites in southern Africa**

**Site**	**An. vector species**	**Fed visually (%)**	**Visually Fed and confirmed molecularly** ^**#**^ **(%)**	**HBI** ^*^	**Visually Unfed but Fed by Kent PCR (%)**	**Visually Unfed, unfed by Kent PCR, Fed by Fornadel PCR (%)**	**Overall PCR confirmed Fed** ^**a**^ **(%)**	**Under-estimation of blood feeding frequency** ^**b**^ **(%)**	**Updated HBI** ^**c**^
**Macha** (n = 643^†^)	*arabiensis* (n = 643)	11.7	7.5	0.96	3.9	11.5	22.1	10.4	0.87
**Nchelenge** Mar-Apr 2012 (n = 411)	*funestus s.s.* (n = 343)	32.4	32.4	1.00	0.86	8.26	38.5	6.1	1.00
	*gambiae s.s.* (n = 36)	25.0	25.0	1.00	3.7	15.4	38.9	13.9	1.00
	*leesoni* (n = 32)	18.8	18.8	0.75	7.7	16.7	37.5	18.7	0.80
**Mutasa** Dec 2012 (n = 84)	*funestus s.s.* (n = 84)	30.5	30.5	0.96	0.0	5.26	35.4	4.9	0.93
	*leesoni* (n = 2)	0.0	0.0	---	0.0	0.0	0.0	---	---

^†^Restricted to specimens detected to be anthropophilic.

^#^Confirmation by both Kent and Fornadel PCRs.

^*^HBI based on visually and molecularly confirmed fed mosquitoes.

^a^Combined results of Kent and Fornadel PCRs run on visually fed and unfed mosquitoes.

^b^Difference in PCR confirmed blood feeding frequency between visually fed mosquitoes only and both visually unfed and fed mosquitoes.

^c^HBI based on molecularly determined fed mosquitoes.

#### Nchelenge district

Of the collected *Anopheles* species, 32.4% (111/343) of *An. funestus*, 25% (9/36) of *An. gambiae,* and 18.8% (6/32) of *An. leesoni* were visually fed and all were molecularly confirmed by both PCR methods, as described by Kent *et al.* [10] and by Fornadel *et al.* [29] (Table 1). Of 126 blood fed *Anopheles*, 111 *An. funestus*, nine *An. gambiae*, and five *An. leesoni* had fed on humans. One specimen of *An. leesoni* had also taken a goat blood meal. The HBIs for both *An. funestus* and *An. gambiae* were 1.00. *An. leesoni* had a lower human blood index of 0.75.

#### Mutasa district

Of the collected *Anopheles* species, 30.5% (25/82) of *An. funestus* were visually fed and all were molecularly confirmed by both PCR methods, as described above [10,29] (Table 1). None of the collected *An. leesoni* were visually fed. Of the 25 blood fed *An. funestus*, 24 had fed on human blood and one had fed on goat. The resulting HBI was 0.96 for *An. funestus*. None of the *An. leesoni* caught were classified as fed.

#### Determination of blood feeding frequency and blood meal source for visually unfed anophelines Choma district

The Kent PCR method revealed 3.9% (22/568) of *An. arabiensis* previously scored visually as unfed had actually taken blood meals (Table 1). It was also found that one *An. arabiensis* had fed on cow and two *An. arabiensis* had fed on goat. There was also one mixed human and dog blood meal detected. Of those classified as unfed by both morphology and both the Kent PCR and the more sensitive Fornadel PCR method revealed that a further 11.5% (63/546) of *An. arabiensis* had actually taken human or other non-human blood meals.

#### Nchelenge district

Of those *Anopheles* that appeared unfed in the field which were subsequently tested for blood meal source by the Kent PCR method, 0.9% (2/232), 3.7% (1/27), and 7.7% (2/26) of unfed *An. funestus s.s., An. gambiae s.s.*, and *An. leesoni* respectively were positive for human and goat blood meals in Nchelenge (Table 1). No other animal host was detected. The Fornadel PCR method revealed that further 8.3% (19/230), 15.4% (4/26), and 16.7% (4/24) of *An. funestus*, *An. gambiae*, and *An. leesoni* respectively that were previously classified as unfed by morphology and the Kent PCR had actually taken human and/or goat blood meals.

#### Mutasa district

Unlike Choma district and Nchelenge district, molecular testing of visually unfed *Anopheles* by the Kent PCR method did not reveal any additional fed mosquitoes. However, the Fornadel PCR revealed that 5.3% (3/57) of the visually unfed *An. funestus* had taken human (2/3) and goat blood meals (1/3) (Table 1). No blood meals were detected by the Kent or the Fornadel PCR methods in the visually unfed *An. leesoni*.

### Overall blood feeding frequency and HBI of *Anopheles*

#### Choma district

Combining the outcomes of the PCRs carried out on anophelines visually scored as fed and unfed revealed that the actual proportions of fed *An. arabiensis* was 22.1% (Table 1). Therefore, visual scoring alone may result in blood feeding rates being underestimated as much as 10.4% compared to PCR detection of blood meals. If determination of host species by Kent PCR was limited to those mosquitoes determined visually as fed, HBI was calculated as 0.96, but if all mosquitoes were analysed using both PCR methodologies, 124/142 *An. arabiensis* had fed on humans, some with mixed animal/human blood meals. This resulted in a reduction in the estimated HBI for *An. arabiensis* to 0.87. Chi-square test results for *An. arabiensis* detected a significant relationship between the visually fed status and the overall PCR confirmed fed status (df = 1; X^2^ = 144.4; *P* < 0.05).

#### Nchelenge district

Of those *Anopheles* specimens classified visually both as fed and unfed, combining the results of the Kent and the Fornadel PCR methods, revealed that the actual proportions of fed *An. funestus s.s*., *An. gambiae s.s*., and *An. leesoni* were 38.5%, 38.9%, and 37.5% respectively in Nchelenge (Table 1). Using just visual assessment of blood feeding status could, therefore, underestimate blood feeding frequency by as much as 18%. After accounting for these blood meals detected in visually unfed *Anopheles*, the HBIs for *An. funestus* and *An. gambiae* remained at 1.00, whereas *An. leesoni* was higher at 0.80. Chi-square test results indicate a significant relationship between the visually fed status and the overall PCR confirmed fed status for all malaria vectors in this area (*An. funestus s.s*.: df = 1, X^2^ = 267.7; *P* < 0.05; *An. gambiae s.s*.: df =1, X^2^ = 21.2; *P* < 0.05; *An. leesoni*: df = 1, X^2^ = 16.2; *P* < 0.05).

#### Mutasa district

The PCR results for both visually fed and unfed *Anopheles* reveals that the overall proportion of fed mosquitoes was 35.4%, suggesting that visual confirmation alone can underestimate blood feeding rates by up to 4.9% (Table 1). After detection of goat blood meals in visually unfed *An. funestus*, the HBI for *An. funestus* reduced to 0.96. Chi-square test analysis revealed a significant relationship between the visually fed status and the overall PCR confirmed fed status for *An. funestus* in this area (df = 1; X^2^ = 168.3; *P* < 0.05).

## Discussion

Through entomological investigations in Choma, *An. arabiensis* has been identified as the primary malaria vector of *P. falciparum* transmission and analysis of blood feeding was restricted to samples identified as this vector. Although this vector is known for its zoophilic behaviour in many parts of Africa, it has been found to be highly anthropophilic in Choma. After molecular testing, the human blood index of *An. arabiensis* decreased due to the identification of blood meals from other animal hosts such as goats and cows in the mosquitoes that were visually unfed. This indicates that *An. arabiensis* takes occasional blood meals on non-human hosts, although many of these may be small meals where the mosquito does not feed to repletion. Although still highly anthropophilic, these previously undetected blood meals dilute the reported rates of anthropophily for this species [29].

In Nchelenge, *An. funestus s.s.* is the most abundant species followed by *An. gambiae s.s*. and *An. leesoni*. Preliminary field collections in the area have confirmed *An. funestus s.s*. and *An. gambiae s.s.* to be the primary and secondary vectors of *P. falciparum* transmission (unpublished data). The malaria parasite has not been detected in *An. leesoni* in Nchelenge. However, the role of *An. leesoni* as a malaria vector in other parts of Africa suggests its potential as a secondary vector in this region and further investigation is required [30]. The human blood indices of both *An. funestus s.s*. and *An. gambiae s.s.* remained the same after molecular testing on all mosquitoes regardless of abdominal status, indicating that they are highly anthropophilic vectors. However, after testing all *An. leesoni* for blood meal host, the updated HBI increased suggesting greater anthropophily than would have been estimated if only visually classified specimens had been analysed.

In Mutasa district, the primary malaria vector of *P. falciparum* is *An. funestus s.s*. (unpublished data). The human blood index of *An. funestus* was reduced slightly after molecular testing of both visually fed and unfed mosquitoes due to detection of additional goat blood meals, but confirms the high anthropophily of this species in eastern Zimbabwe. None of the collected *An. leesoni* were visually fed or molecularly confirmed as fed. As a result, it was not possible to determine the blood meal source and resulting HBI of this potential vector species.

For basic malaria vector studies, identifying the host of mosquito blood meals is a crucial step in estimating vector transmission potential and intensity of malaria found in an area. When mosquito collections take place in the field, it is common practice to have trained personnel identify each mosquito and also classifies the abdominal status by morphology. Once in a laboratory setting, normally only those mosquitoes labelled as “fed” are separated and tested for blood meal host, and even then only if the infrastructure and financial support exists to conduct these assays. However, in this study, a significant proportion of visually classified “unfed” mosquitoes had detectable blood meals by PCR methods. The Kent and the Fornadel PCR protocols used in this study amplify different portions of the *cytochrome b* gene, but the Fornadel PCR is more sensitive by being able to detect blood meals up to 60 hours post-feeding in laboratory experiments [26,29]. A large proportion of visually “unfed” mosquitoes were found to be blooded by the Kent PCR and a further number were found to be fed by the Fornadel PCR assay. By only testing the visually “fed” mosquitoes for blood meal host identification, the true proportion of fed mosquitoes in a collection may be underestimated by as much as 18%. This trend was evident in three epidemiological distinct sites in southern Africa. Conversely, it was also observed in the Choma site that a small proportion of the visually fed mosquitoes did not contain a blood meal as determined by the Kent and the Fornadel PCR protocols. This may occur because of incorrect classification of the specimen, desiccation of specimens resulting in dark pigmentation that can be mistaken for blood in mosquitoes, specimens with enlarged abdomens may actually be gravid or half gravid, or contain a sugar meal. It may also be due to the inherent limitation of the PCR assays used [26,29].

The molecular confirmation of “unfed” mosquitoes actually being fed may be due to several reasons. Firstly, it may indicate partial feeding behaviour, resulting in a blood meal size that is undetectable by the human eye. In the field, host defensive behaviours can interrupt a mosquito’s ability to reach repletion [31]. Previous field studies using unrestrained hosts in stable traps found that a large proportion of *Culex tarsalis* mosquitoes were attracted to the bait, but took partial or no blood meals [32,33]. Similarly, laboratory-reared mosquitoes also experienced decreased feeding success due to defensive host behaviours [31,34,35]. Another factor that may result in partial or reduced blood feeding is vector control; at all three sites of this study, vector control such as LLINs and/or IRS have been implemented in response to which mosquitoes may limit their duration of contact with a host to avoid insecticides [30].

Consequently, mosquitoes may be unable to reach repletion during feeding and must take multiple blood meals during a gonotrophic cycle. This has important implications for estimating vector potential and malaria transmission risk in endemic areas [15,31,36]. This study was not designed to assess feeding behaviour pre- and post-intervention. Clearly, further research needs to be done to ascertain the extent of anopheline partial blood feeding behaviour in Africa. In addition to partial feeding, mosquitoes may have undergone partial digestion of the blood meal such that the volume remaining is not easily detectable by eye. Visual assessment of blood feeding status may also be hindered by sample condition such as desiccation or damage. Additionally, personnel must be trained to correctly assess the abdominal status.

Overall, if all collected mosquitoes are not tested for blood meal host, then the proportion fed, HBI, and even EIR may be miscalculated and the accuracy of vector studies may be diminished. The proportion fed in a collection can be an important component for testing and evaluating vector control interventions such as LLINs, IRS, or spatial repellents. Efficacy may be determined by observing a reduction in feeding behaviour by vectors as well as changes in other parameters such as deterrency, entry/exit behaviour and mortality rates [37-41]. The HBI, a component of vector capacity, provides crucial information about mosquito feeding patterns and vector-host association [42]. Additionally an incorrect estimate of the number of fed mosquitoes can lead to a miscalculation of biting rates and therefore EIR. The relationship between EIR and malaria prevalence is not direct, but EIR can range from 0 to 1500 infective bites per person per year in endemic parts of Africa [43,44]. Thus, it can be a useful measurement in defining malaria endemicity and transmission intensity [43,45]. Accuracy in the calculations for HBI and EIR are essential for defining malaria transmission and dynamics in affected locales [46], and for guiding appropriate control strategies and assessing their effectiveness. Based on this study, it is predicted that in areas with highly anthropophilic vectors such as Nchelenge and Mutasa Districts, the HBI and EIR will show little or no change when testing for blood meal source in all mosquitoes. However, in areas with both anthropophilic and zoophilic vectors such as Choma, testing the blood meal source in all mosquitoes could affect both the HBI and EIR.

## Conclusions

The present study illustrates the importance of testing morphologically unfed and fed mosquitoes for identification of host blood meal. By not testing all mosquitoes in a collection, inaccurate measurement of the HBI and even the EIR may result. Misestimation of the HBI occurred when restricting testing to only those visually fed, even at sites with very different vector compositions and epidemiology. Both the HBI and EIR contribute to the understanding of malaria transmission intensity by *Anopheles* mosquitoes; these parameters not only help direct control efforts, but also provide tools for surveillance by assessing potential changes in foraging behaviour in response to vector control or other ecological changes. The visually unfed mosquitoes that have detectable blood meals by molecular methods may suggest partial feeding behaviour, a response to vector control measures, partial blood meal digestion that is undetectable by eye, or errors in interpreting unfed or fed abdomens by personnel. Although performing molecular techniques to identify host blood meals of all morphologically fed and unfed mosquitoes is ideal for increased accuracy in measurements of anopheline foraging behaviour and estimation of EIR, it may pose a challenge for resource-limited countries to be able to perform such extensive testing. As a result, it is suggested that sub-sampling and extrapolation can be used for morphological and molecular determination of host blood meal in order to more accurately characterize mosquito feeding behaviour in malaria endemic areas.

## References

[1] WHO (2013). World Malaria Report.

[2] Garrett-Jones C (1964). Prognosis for interruption of malaria transmission through assessment of the mosquito’s vectorial capacity. Nature.

[3] Garrett-Jones C, Shidrawi GR (1969). Malaria vectorial capacity of a population of *Anopheles gambiae*: an exercise in epidemiological entomology. Bull World Health Organ.

[4] Chitnis N, Smith T, Steketee R (2008). A mathematical model for the dynamics of malaria in mosquitoes feeding on a heterogeneous host population. J Biol Dyn.

[5] James S, Takken W, Collins FH, Gottlieb M (2014). Needs for monitoring mosquito transmission of malaria in a pre-elimination world. Am J Trop Med Hyg.

[6] Kiware SS, Chitnis N, Moore SJ, Devine GJ, Majambere S, Merrill S (2012). Simplified models of vector control impact upon malaria transmission by zoophagic mosquitoes. PLoS One.

[7] Smith DL, Dushoff J, McKenzie FE (2004). The risk of a mosquito-borne infection in a heterogeneous environment. PLoS Biol.

[8] Adeleke MA, Mafiana CF, Idowu AB, Sam-Wobo SO, Idowu OA (2010). Population dynamics of indoor sampled mosquitoes and their implication in disease transmission in Abeokuta, south-western Nigeria. J Vector Borne Dis.

[9] Fontenille D, Lochouarn L, Diatta M, Sokhna C, Dia I, Diagne N (1997). Four years’ entomological study of the transmission of seasonal malaria in Senegal and the bionomics of *Anopheles gambiae* and *A. arabiensis*. Trans R Soc Trop Med Hyg.

[10] Kent RJ, Thuma PE, Mharakurwa S, Norris DE (2007). Seasonality, blood feeding behavior, and transmission of *Plasmodium falciparum* by *Anopheles arabiensis* after an extended drought in southern Zambia. Am J Trop Med Hyg.

[11] Massebo F, Balkew M, Gebre-Michael T, Lindtjorn B (2013). Blood meal origins and insecticide susceptibility of *Anopheles arabiensis* from Chano in South-West Ethiopia. Parasit Vectors.

[12] Muriu SM, Muturi EJ, Shililu JI, Mbogo CM, Mwangangi JM, Jacob BG (2008). Host choice and multiple blood feeding behaviour of malaria vectors and other anophelines in Mwea rice scheme, Kenya. Malar J.

[13] Gillies T, Coetzee M (1987). A Supplement to the Anophelinae of Africa South of the Sahara.

[14] Mwangangi JM, Mbogo CM, Orindi BO, Muturi EJ, Midega JT, Nzovu J (2013). Shifts in malaria vector species composition and transmission dynamics along the Kenyan coast over the past 20 years. Malar J.

[15] Norris LC, Fornadel CM, Hung WC, Pineda FJ, Norris DE (2010). Frequency of multiple blood meals taken in a single gonotrophic cycle by *Anopheles arabiensis* mosquitoes in Macha, Zambia. Am J Trop Med Hyg.

[16] Russell TL, Beebe NW, Cooper RD, Lobo NF, Burkot TR (2013). Successful malaria elimination strategies require interventions that target changing vector behaviours. Malar J.

[17] Lindsay SW, Snow RW, Broomfield GL, Janneh MS, Wirtz RA, Greenwood BM (1989). Impact of permethrin-treated bednets on malaria transmission by the *Anopheles gambiae* complex in The Gambia. Med Vet Entomol.

[18] Mathenge EM, Gimnig JE, Kolczak M, Ombok M, Irungu LW, Hawley WA (2001). Effect of permethrin-impregnated nets on exiting behavior, blood feeding success, and time of feeding of malaria mosquitoes (Diptera: Culicidae) in western Kenya. J Med Entomol.

[19] N’Guessan R, Corbel V, Akogbeto M, Rowland M (2007). Reduced efficacy of insecticide-treated nets and indoor residual spraying for malaria control in pyrethroid resistance area, Benin. Emerg Infect Dis.

[20] Snow RW, Lindsay SW, Hayes RJ, Greenwood BM (1988). Permethrin-treated bed nets (mosquito nets) prevent malaria in Gambian children. Trans R Soc Trop Med Hyg.

[21] Burkot TR, Graves PM (1995). The value of vector-based estimates of malaria transmission. Ann Trop Med Parasitol.

[22] Animut A, Balkew M, Gebre-Michael T, Lindtjorn B (2013). Blood meal sources and entomological inoculation rates of anophelines along a highland altitudinal transect in south-central Ethiopia. Malar J.

[23] WHO (2003). Malaria Entomology and Vector Control: Learner’s Guide. Trial Edition HIV/AIDS, Tuberculosis and Malaria, Roll Back Malaria.

[24] Amek N, Bayoh N, Hamel M, Lindblade KA, Gimnig JE, Odhiambo F (2012). Spatial and temporal dynamics of malaria transmission in rural Western Kenya. Parasit Vectors.

[25] Moss WJ, Norris DE, Mharakurwa S, Scott A, Mulenga M, Mason PR (2012). Challenges and prospects for malaria elimination in the Southern Africa region. Acta Trop.

[26] Kent RJ, Norris DE (2005). Identification of mammalian blood meals in mosquitoes by a multiplexed polymerase chain reaction targeting cytochrome B. Am J Trop Med Hyg.

[27] Koekemoer LL, Kamau L, Hunt RH, Coetzee M (2002). A cocktail polymerase chain reaction assay to identify members of the *Anopheles funestus* (Diptera: Culicidae) group. Am J Trop Med Hyg.

[28] Scott JA, Brogdon WG, Collins FH (1993). Identification of single specimens of the *Anopheles gambiae* complex by the polymerase chain reaction. Am J Trop Med Hyg.

[29] Fornadel CM, Norris DE (2008). Increased endophily by the malaria vector *Anopheles arabiensis* in southern Zambia and identification of digested blood meals. Am J Trop Med Hyg.

[30] Sokhna C, Ndiath MO, Rogier C (2013). The changes in mosquito vector behaviour and the emerging resistance to insecticides will challenge the decline of malaria. Clin Microbiol Infect.

[31] Klowden MJ, Lea AO (1979). Effect of defensive host behavior on the blood meal size and feeding success of natural populations of mosquitoes (Diptera: Culicidae). J Med Entomol.

[32] Blackmore JS, Dow RP (1958). Differential feeding of *Culex tarsalis* on nestling and adult birds. Mosq News.

[33] Nelson RL, Tempelis CH, Reeves WC, Milby MM (1976). Relation of mosquito density to bird: mammal feeding ratios of *Culex tarsalis* in stable traps. Am J Trop Med Hyg.

[34] Edman JD, Kale HW (1971). Host behavior: its influence on the feeding success of mosquitoes. Ann Entomol Soc Am.

[35] Edman JD, Webber LA, Kale HW (1972). Effect of mosquito density on the interrelationship of host behavior and mosquito feeding success. Am J Trop Med Hyg.

[36] Klowden MJ, Lea AO (1978). Blood meal size as a factor affecting continued host-seeking by *Aedes aegypti* (L.). Am J Trop Med Hyg.

[37] Bugoro H, Cooper RD, Butafa C, Iro’ofa C, Mackenzie DO, Chen CC (2011). Bionomics of the malaria vector *Anopheles farauti* in Temotu Province, Solomon Islands: issues for malaria elimination. Malar J.

[38] Guillet P, N’Guessan R, Darriet F, Traore-Lamizana M, Chandre F, Carnevale P (2001). Combined pyrethroid and carbamate ‘two-in-one’ treated mosquito nets: field efficacy against pyrethroid-resistant *Anopheles gambiae* and *Culex quinquefasciatus*. Med Vet Entomol.

[39] Malima R, Tungu PK, Mwingira V, Maxwell C, Magesa SM, Kaur H (2013). Evaluation of the long-lasting insecticidal net Interceptor LN: laboratory and experimental hut studies against anopheline and culicine mosquitoes in northeastern Tanzania. Parasit Vectors.

[40] Malima RC, Oxborough RM, Tungu PK, Maxwell C, Lyimo I, Mwingira V (2009). Behavioural and insecticidal effects of organophosphate-, carbamate- and pyrethroid-treated mosquito nets against African malaria vectors. Med Vet Entomol.

[41] McCann RS, Ochomo E, Bayoh MN, Vulule JM, Hamel MJ, Gimnig JE (2014). Reemergence of *Anopheles funestus* as a vector of *Plasmodium falciparum* in western Kenya after long-term implementation of insecticide-treated bed nets. Am J Trop Med Hyg.

[42] Lefevre T, Gouagna LC, Dabire KR, Elguero E, Fontenille D, Renaud F (2009). Beyond nature and nurture: phenotypic plasticity in blood-feeding behavior of *Anopheles gambiae* s.s. when humans are not readily accessible. Am J Trop Med Hyg.

[43] Beier JC, Oster CN, Onyango FK, Bales JD, Sherwood JA, Perkins PV (1994). *Plasmodium falciparum* incidence relative to entomologic inoculation rates at a site proposed for testing malaria vaccines in western Kenya. Am J Trop Med Hyg.

[44] Elissa N, Migot-Nabias F, Luty A, Renaut A, Toure F, Vaillant M (2003). Relationship between entomological inoculation rate, *Plasmodium falciparum* prevalence rate, and incidence of malaria attack in rural Gabon. Acta Trop.

[45] Okello PE, Van Bortel W, Byaruhanga AM, Correwyn A, Roelants P, Talisuna A (2006). Variation in malaria transmission intensity in seven sites throughout Uganda. Am J Trop Med Hyg.

[46] Kelly-Hope LA, McKenzie FE (2009). The multiplicity of malaria transmission: a review of entomological inoculation rate measurements and methods across sub-Saharan Africa. Malar J.

